# Reply to: ‘Melanoma diagnosis at a specialist dermatology practice without the use of photographic surveillance’

**DOI:** 10.1111/ajd.14087

**Published:** 2023-05-25

**Authors:** Nikki R. Adler, Anne E. Cust, Victoria J. Mar, Linda K. Martin, Pascale Guitera

**Affiliations:** ^1^ Victorian Melanoma Service Alfred Hospital Melbourne Victoria Australia; ^2^ School of Public Health and Preventive Medicine Monash University Melbourne Victoria Australia; ^3^ The Daffodil Centre The University of Sydney, a Joint Venture with Cancer Council NSW Sydney New South Wales Australia; ^4^ Melanoma Institute Australia The University of Sydney Sydney New South Wales Australia; ^5^ Faculty of Medicine & Health University of New South Wales Sydney New South Wales Australia; ^6^ Faculty of Medicine and Health The University of Sydney Sydney Australia; ^7^ Sydney Melanoma Diagnostic Centre Royal Prince Alfred Hospital Camperdown New South Wales Australia

Brown et al.'s^1^ study of primary melanomas diagnosed at a private dermatology practice by full skin examination with dermoscopy, but without total body photography (TBP) or sequential digital dermoscopic imaging (SDDI), reported a ratio of in situ to invasive melanoma of 4.6:1, which the authors infer is higher than previous studies utilising TBP/SDDI.

When assessing the quality of melanoma screening, the benign to malignant ratio of excised lesions, which was not reported by Brown et al.,^1^ is more important than the ratio of in situ to invasive disease as it provides more accurate assessment of screening effectiveness. A melanoma screening quality framework is needed, which includes who to screen (targeted screening), how to screen (which methodologies) and how it should be evaluated.

We do not advocate for general population skin cancer screening, nor the use of melanoma surveillance photography in low‐risk patients. In Brown's study, only 14% of patients had melanoma risk factors extracted and among these, only 25% met our high‐risk criteria.^2^ This patient cohort is not comparable with those of Guitera et al, where photographic surveillance was used for detection of subsequent primary melanomas.^2^ Prospective, controlled studies are required to accurately assess melanoma surveillance photography in reducing unnecessary biopsies and enhancing early detection. We look forward to results of the IMAGE trial of high‐risk patients, in which a reduction in benign biopsies is a primary objective.^3^

We are concerned by the biopsy practices in Brown's study, with over half of invasive melanomas diagnosed by shave procedures and 4.3% with deep margin transection.^1^ Compared with elliptical excision, shave procedures are more likely to transect the tumour base and reduce capacity to accurately assess tumour depth for prognostication, staging and treatment planning.^4^, ^5^ The Australian Cancer Council Guidelines recommend complete excision with 2 mm margin for suspicious pigmented lesions.^4^

The high rates of in situ melanoma in Brown's report may be partly attributed to overdiagnosis. In Brown's study, histopathological results were obtained from a single pathology provider.^1^ A study by Elmore and colleagues demonstrated that pathologists' diagnoses within the spectrum from moderately dysplastic naevi to early‐stage invasive melanoma are neither reproducible nor very accurate.^6^ The higher rates of lentigo maligna in Queensland may also contribute to the reported high ratio of in situ to invasive disease.

We disagree that targeted screening with the use of photographic surveillance for high‐risk patients is ‘dangerous’ and ‘risks patient safety.’^1^ This demonstrates a misunderstanding of photographic monitoring. Monitoring is not recommended for lesions with specific melanoma dermoscopic features or nodules.^7^ SSDI/TBP can identify featureless melanoma not diagnosable with clinical examination and dermoscopy. The frequency of invasive featureless melanoma of small diameter identified purely from TBP is noteworthy.^8^ The diagnosis of such lesions is unable to be identified in Brown's study, which does not include information on the number of screening visits per patient, time interval between first screening and melanoma diagnosis, nor number of biopsies yielding benign diagnoses in their entire patient cohort during this period. Despite these factors, there were more melanomas diagnosed than appointments,^1^ which seems unexpected.

## FUNDING INFORMATION

AEC is supported by a NHMRC Investigator Grant (#2008454). LKM is supported by the Warwick L Morison Professorship, UNSW.

## CONFLICT OF INTEREST STATEMENT

NA, AEC, VM and PG are members of the Australian Cancer Research Foundation funded Australian Centre of Excellence for Melanoma Imaging and Diagnosis consortium. VM has received speaker fees from Novartis, Bristol Myers Squibb, Merck and Janssen, and conference sponsorship from L'Oreal. PG has received speaker fees from L'Oreal and honoraria form Metaoptima, PTY. NA is an Editorial Board member of Australasian Journal of Dermatology and co‐author of this article. To minimise bias, they were excluded from all editorial decision‐making related to the acceptance of this article for publication.
